# Suicide Prevention and the Sustainable Development Goals: The Case of Bangladesh

**DOI:** 10.1002/puh2.70382

**Published:** 2026-09-21

**Authors:** Anisur Rahman Khan, S. M. Yasir Arafat, Najuwa Arendse

**Affiliations:** ^1^ Department of Sociology East West University Dhaka Bangladesh; ^2^ Institute for Social and Health Sciences (ISHS) University of South Africa Johannesburg South Africa; ^3^ Biomedical Research Foundation Dhaka Bangladesh; ^4^ Department of Psychiatry Bangladesh Specialized Hospital Dhaka Bangladesh; ^5^ Violence, Injury and Social Asymmetries Research Unit University of South Africa and South African Medical Research Council Cape Town South Africa

**Keywords:** Bangladesh, mental health, suicide prevention, Sustainable Development Goals (SDGs), World Health Organization (WHO)

## Abstract

Every year, more than 720,000 people worldwide die by suicide, with a striking 73% of these deaths occurring in low‐ and middle‐income countries (LMICs). The United Nations Sustainable Development Goal (SDG) Indicator 3.4.2 aims to reduce suicide mortality by one‐third, yet Bangladesh faces substantial challenges in meeting this benchmark. Although the official 2021 World Health Organization (WHO) estimate places the national suicide rate at 2.8 per 100,000, independent sources suggest a much higher burden of annual deaths—often obscured by misclassification and underreporting. Despite recent advances, such as the National Mental Health Policy (2022) and the National Mental Health Strategic Plan (2020–2030), which targets a 5% reduction by 2025, implementation has stalled. Key obstacles persist, including the ongoing criminalization of suicide attempts, the absence of centralized surveillance data, and lack of a dedicated national prevention strategy. This commentary analyzes the intersection between global SDG targets and the Bangladeshi context, advocating for a multisectoral public health approach. To achieve measurable progress, we emphasize the need for decriminalization, restriction of access to lethal means, responsible media engagement, and increased financial investment in community‐ and school‐based interventions.

## 1 Introduction

Suicide is a global public health crisis and one of the leading causes of mortality, accounting for more than 720,000 deaths each year [1]. The risk for suicide is multifaceted and varies considerably across regions and countries, where 73% of these deaths occur in low‐ and middle‐income countries (LMICs) [1, 2, 3]. Bangladesh, an LMIC South Asian country, reported 4714 suicide deaths in 2021 and an estimated rate of 7.8 per 100,000 population [1, 4, 5], which is lower than the global average (8.9 per 100,000), the Southeast Asia regional rate (10.1 per 100,000), and neighboring LMICs, such as India, Pakistan, Sri Lanka, and Nepal [1]. In Bangladesh, suicide does not receive adequate attention as a public health issue due to the absence of a national suicide surveillance system, resulting in an incomplete understanding of its epidemiology [6]. Biological, psychosocial, and environmental factors all play significant roles in influencing suicide risk [7]. Unlike in the Americas and Europe, where middle‐aged and older men—especially those experiencing mental illness, unemployment, or social isolation—face the highest risk, the Bangladeshi population at risk skews younger. In Bangladesh, individuals under 30, women, married persons, students, and homemakers are particularly vulnerable [4, 8, 9]. More specifically, the variations in suicide risk in Bangladesh have been attributed to diverse socioeconomic and cultural factors, such as unemployment, poverty, domestic violence, and untreated mental health conditions, like depression and anxiety [10]. Structural barriers in mental health support are significant risk factors for suicidal behavior in Bangladesh. The *National Mental Health Survey of Bangladesh* (2018–2019) estimated that 18.7% of adults experienced a mental disorder in the previous year, with depression and anxiety being the most common [11]. However, most do not receive treatment due to limited services, financial barriers, stigma, and a shortage of mental health professionals [12]. Independent studies indicate that suicide rates are higher in rural areas compared to urban regions in Bangladesh, with hanging and poisoning being the predominant methods. Underreporting and misclassification of suicide deaths remain significant issues, primarily driven by criminalization, pervasive stigma, and sociocultural barriers [6, 13]. Preventing suicide is vital not only for protecting individuals and families but also for promoting societal well‐being, strengthening the health care system, and supporting the broader economy [14]. The *United Nations (UN) Sustainable Development Goals* (SDGs) specifically target the reduction of suicide mortality, as highlighted in several international documents and action plans. However, efforts and progress toward achieving this goal remain inadequate [2, 14, 15, 16, 17, 18].

Bangladesh has not yet developed a strong public health response for suicide prevention, leading to ongoing uncertainty about progress on the SDG indicator for suicide reduction. Addressing this issue will require coordinated efforts across society, including widespread awareness campaigns, policy interventions, institutional and financial support, and stronger professional engagement from relevant sectors. This commentary aims to explore the relationship between suicide reduction as an SDG target and the context of Bangladesh, with a particular focus on potential policy and programmatic directions.

## 2 SDG and Suicide: The Interconnectedness

**FIGURE 1:**
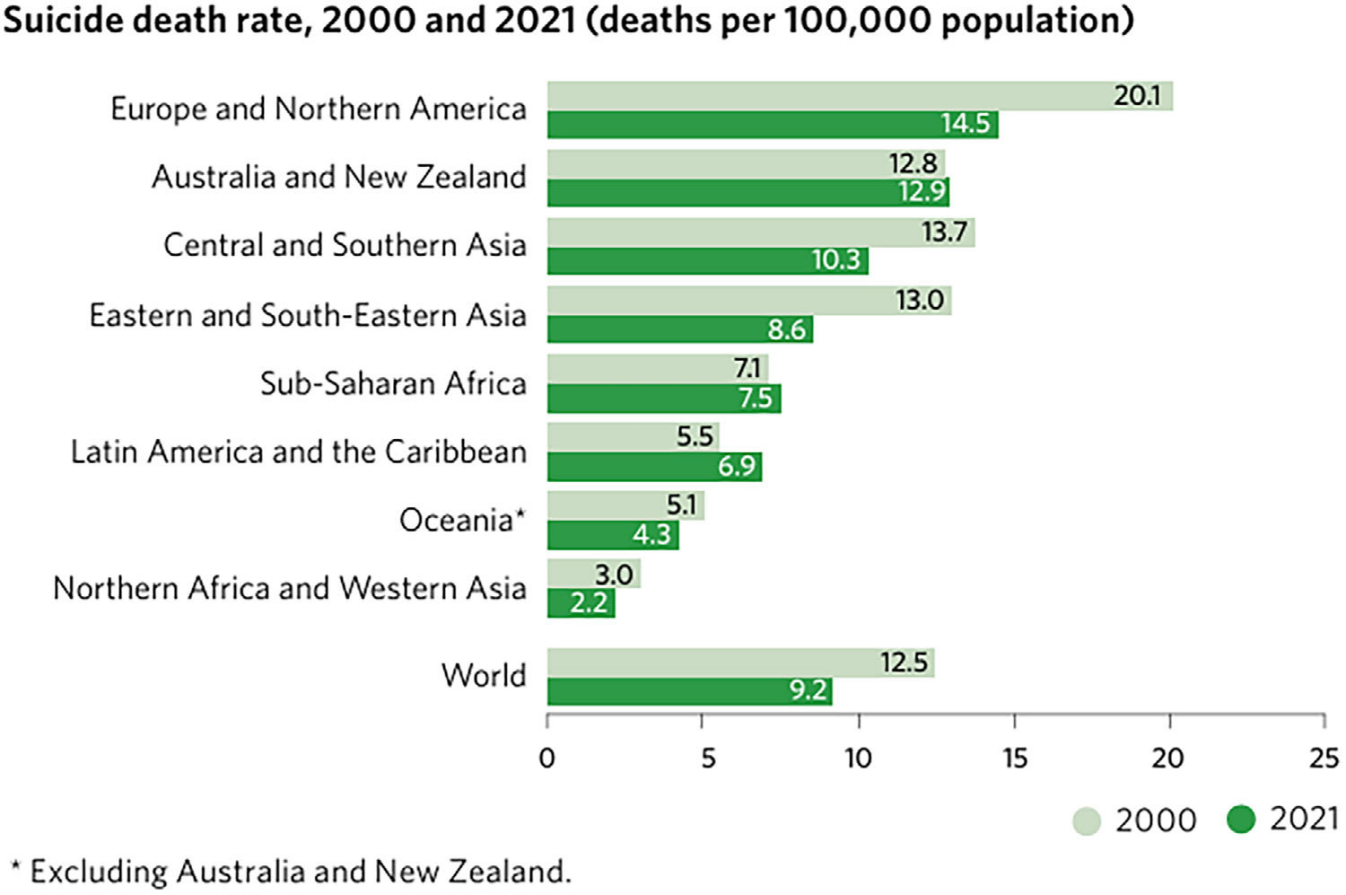
Suicide rates in 2000 and 2021 in different regions [20].

In 2015, the UN launched the SDGs, building on the foundation of the Millennium Development Goals (MDGs). Goal 3, “Ensure healthy lives and promote well‐being for all at all ages,” features Target 3.4, which seeks to reduce premature mortality from noncommunicable diseases by one‐third. Notably, this target emphasizes the promotion, prevention, and treatment of mental health problems, with suicide mortality identified as Indicator 3.4.2 [15, 19]. As an SDG indicator, suicide mortality is defined as the number of suicide deaths in a year divided by the total population and multiplied by 100,000 [20]. The United Nations Sustainable Development Goals Report 2025 indicates that, although the global crude suicide rate declined from 12.5 to 9.2 per 100,000 population between 2000 and 2021, progress has been inconsistent across regions and contexts [21]. The following trend illustrates disparities in crude suicide rates across different regions (Figure 1).

Overall, suicide rates have declined significantly over the past two decades, with the UN reporting substantial decreases in Europe, North America, and Asia (Figure 1). According to the World Health Organization (WHO), suicide rates in Bangladesh have steadily decreased over the past decade, dropping from 6.6 per 100,000 in 2012 to 2.8 per 100,000 in 2021 [1], even though no clear preventive strategies were implemented. These estimates contradict the field‐level surveys and police data, raising concerns about data quality, misclassification, and underreporting [4, 9]. Although the global community is currently falling short, not only in meeting the SDG target for suicide reduction but also in adequately evaluating whether individuals have equitable access to the services that could prevent loss of life [22]. This raises uncertainty about sustaining progress toward achieving the goal of a one‐third reduction in suicide rates [3].

## 3 Major Global Strategies and Actions for Suicide Prevention

For decades, various international agencies have offered suicide prevention guidance and frameworks to nation–states, even before the introduction of the UN SDGs. Specifically, the WHO has led a comprehensive international policy response, promoting evidence‐based strategies and actions to reduce suicide and establishing measurable targets [23]. In 2003, the World Suicide Prevention Day (WSPD), in collaboration with the WHO, launched a major advocacy and awareness‐raising initiative that sought to engage national organizations, governments, and the public with the unified message that suicide is preventable [24]. The WHO Mental Health Action Plan 2013–2020, adopted in 2013, saw member states reaffirmed their commitment to achieve the global target of reducing the suicide rate by 10% by 2020 [25, 26]. These developments were followed by the release of three major reports and the launch of the WHO LIVE LIFE initiative, which together provided a stronger foundation for global suicide prevention efforts.

A significant milestone in advancing the global suicide prevention agenda was the publication of the WHO report, Preventing Suicide: A Global Imperative, in 2014 [16, 25]. This comprehensive resource offers an extensive global knowledge base on suicide and suicide attempts, as along with actionable guidance to help countries advance their suicide prevention efforts [16]. Nation–states have been urged to adopt a broad, multisectoral public health strategy that effectively engages stakeholders across sectors, taking into account available resources and cultural contexts. The report also identifies major risk factors at the individual, relationship, community, and societal levels, and links them to three types of interventions: universal, selective, and indicated [16]. National Suicide Prevention Strategies: Progress, Examples and Indicators was introduced to assist countries in sustaining progress in suicide prevention and to encourage governments and policymakers to develop or update national strategies that actively involve local communities [14]. The report emphasized the importance of implementing a comprehensive, multisectoral approach and assessing measures to overcome existing barriers. The critical role of governments in actively engaging in suicide prevention efforts is also highlighted [14]. Preventing Suicide: A Community Engagement Toolkit [17] provides a practical resource for initiating suicide prevention at the community level. The toolkit promotes a participatory, bottom‐up approach that emphasizes the shared ownership of activities by local stakeholders. In addition, guidance is provided on engaging diverse actors to identify priorities and design interventions tailored to the local context. It offers step‐by‐step instructions for community‐based suicide prevention in six key areas: initial preparation, initiating the conversation at the first meeting, creating a community action plan, ongoing media engagement, monitoring and evaluating the community action plan, and conducting community feedback meetings. Each section outlines strategies for advancing community engagement and highlights tools to help develop a context‐specific suicide prevention action plan [17].

The WHO's [18] LIVE LIFE: An Implementation Guide for Suicide Prevention in Countries offers a strategic framework for suicide prevention. The LIVE LIFE model provides global and national policy directions to advance implementation research and practice in suicide prevention [27]. This framework enables countries to develop comprehensive national suicide prevention strategies. The LIVE LIFE framework is structured around two main components: (a) core pillars and (b) suicide prevention interventions. The core pillars include situational analysis, multisectoral collaboration, awareness‐raising and advocacy, capacity‐building, financing, and surveillance, monitoring, and evaluation. The intervention components focus on restricting access to means of suicide, engaging with media for responsible reporting, promoting socio‐emotional life skills among young people, and ensuring early identification and support for individuals affected by suicide and self‐harm. The implementation of these interventions is reinforced and guided by the core pillars [18].

The documents and action plans outlined above provide essential guidance for formulating effective suicide prevention strategies and achieving the SDG target. However, because suicidal behavior is complex and influenced by multiple factors, effective prevention requires a comprehensive range of strategies and actions [23]. Scholars highlight the need for coordinated and collaborative suicide prevention strategies that address both population‐ and individual‐level factors [28]. They also advocate for a whole‐of‐government strategy involving cross‐sectoral action, social participation, and community empowerment [29], as well as the implementation of universal, selective, and indicated interventions [30]. Although Bangladesh's 2018 Mental Health Act did not include suicide prevention, the 2022 Mental Health Policy recognizes the need for suicide research, emphasizes the importance of reliable data, promotes suicide awareness initiatives and preventative measures, and highlights the significance of understanding relevant risk factors [31].

Although international best practices call for comprehensive suicide prevention—through national strategies, surveillance systems, accessible mental health care, means restriction, and community‐based interventions—Bangladesh still faces significant implementation gaps. In this regard, Arafat [32] highlights the lack of a national suicide prevention program, a unified suicide database, and sufficient clinical services as key barriers to effective prevention. Ecological analyses indicate that suicide in Bangladesh results from a complex interplay of individual, family, community, and societal factors. This suggests that prevention efforts limited to psychiatric treatment alone are likely insufficient [33, 34]. These findings suggest that the adaptation of international best practices will require culturally tailored interventions that address gender inequality, family conflict, stigma, and barriers to mental health services, alongside efforts to strengthen the healthcare system.

## 4 Suicide in Bangladesh: Progress and Challenges Toward the SDGs

Bangladesh has made significant progress in a number of SDGs, including SDG 1 (No Poverty), SDG 4 (Quality Education), SDG 5 (Gender Equality), SDG 8 (Decent Work and Economic Growth), SDG 10 (Reduced Inequalities), SDG 11 (Sustainable Cities and Communities), and SDG 13 (Climate Action) [35]. However, Bangladesh continues to lag in implementing suicide prevention measures. The country still lacks a national suicide prevention strategy and has not established a suicide surveillance system, both of which are essential to address the population's mental health vulnerabilities [36].

### 4.1 Available Suicide Preventive Measures

Three notable suicide prevention initiatives are currently underway in Bangladesh: *Kaan Pete Roi* (KPR) (private national crisis helpline), the National Emergency Service (NES)‐999, and legislation banning the agricultural use of WHO Class I highly hazardous pesticides (HHPs). However, their effectiveness varies, and significant gaps persist. The first private suicide‐prevention helpline, KPR, was launched in 2013 and has gradually expanded its services, demonstrating the potential value of crisis intervention and emotional support. KPR is the country's only dedicated telephone‐based crisis helpline for emotional support and suicide prevention [33]. This Bangladeshi service has demonstrated promising outcomes, gaining widespread recognition for its significant contribution to suicide prevention. By January 2022, KPR had intervened in and prevented 1492 suicide cases [33]. NES provides only partial support for suicide crises as part of the broader NES, whereas KPR operates on a limited 12‐h daily schedule [33]. Although these interventions successfully de‐escalated calls, this success cannot be equated with lives that were saved, and, subsequently, there is insufficient evidence to provide measurement thereof. Relative to other countries with well‐established helplines, Bangladesh demonstrates lower utility rates of this service that is attributed to the cost and accessibility, low outreach, and limited availability, among others. For example, in the United States, the Suicide and Crisis Helpline reported 8.05 million contacts [37].

Second, the NES‐999 was launched on December12, 2017 to respond to a broad range of emergencies, including suicide crises. In its first 5 years, the service helped rescue approximately 1500 individuals from suicide attempts. However, over 1100 people still died by suicide because responders could not reach them in time [33]. Although these figures demonstrate the potential life‐saving role of NES‐999, they also reveal major operational challenges. Delays in response times, difficulties in locating individuals in crisis, transportation barriers, inadequate emergency infrastructure, and limited coordination between emergency responders and mental health services all reduce the system's effectiveness. A general emergency service mainly intervenes after a crisis has escalated, rather than offering ongoing risk assessment, follow‐up care, or preventive mental health support. The high number of suicides reported despite emergency notification underscores that crisis response alone is not enough. Effective suicide prevention requires an earlier identification of at‐risk individuals, robust community‐based interventions, and accessible mental health services. Lastly, as a means of restricting access to lethal means, the Bangladeshi government introduced legislation banning the agricultural use of WHO Class I HHPs [38]. However, evidence suggests that enforcement remains inconsistent. In many rural areas, highly toxic pesticides continue to be accessible and are widely used in agriculture, maintaining a significant risk factor for self‐poisoning [39, 40]. Consequently, pesticide poisoning remains a major clinical and public health concern, indicating that legislative action alone is insufficient without effective monitoring, enforcement, safe storage practices, and community education.

### 4.2 Strategies and Policy Initiatives

Bangladesh has adopted the *National Mental Health Policy* (2022) and the *National Mental Health Strategic Plan* (2020–2030), both of which recognize suicide prevention as a critical public health priority. The *National Mental Health Policy* (2022) emphasizes on three key areas: (a) developing an inclusive national suicide prevention strategy, (b) increasing public awareness, and (c) promoting systematic research to better understand and prevent suicide and suicide attempts [31]. Similarly, the *National Mental Health Strategic Plan* (2020–2030) sets a national target of reducing suicide deaths by 5% by 2025. A central objective is to reduce the risk and incidence of suicide and suicide attempts by increasing community awareness and promoting help‐seeking behaviors. To achieve this, the plan highlights several key actions: conducting national‐level research, establishing a national referral system, developing a national strategy on substance misuse and suicidal behavior, expanding support services, restricting access to means of suicide (such as hazardous pesticides), decriminalizing suicide, improving referral to health services, ensuring emergency services for suicide attempts, implementing a national suicide prevention program, providing gatekeeper training, operating a crisis helpline, offering postvention services, and promoting responsible media reporting [41].

### 4.3 Policy Practice Gap

Although Bangladesh's *National Mental Health Policy* (2022) and the *National Mental Health Strategic Plan* (2020–2030) outline promising actions and commitments, their implementation on the ground remains limited, with little evidence of coordinated nationwide efforts [32]. Although the *National Mental Health Strategic Plan* aimed to reduce suicide by 5%, Bangladesh does not have regular, publicly available and periodic data, making it difficult to track progress. Suicide continues to be a significant public health concern and is frequently reported in mainstream media [42]. Gaps persist between policy commitments and actual implementation, particularly in data collection, service delivery, intersectoral coordination, and the evaluation of prevention initiatives.

### 4.4 The Challenges

Bangladesh does not have a national suicide surveillance system or a coordinated support network. Suicide and attempted suicide are still criminalized, and both are further complicated by social stigma, cultural taboos, and low public awareness [43]. Under Section 306 of the Penal Code of 1860, police can arrest and courts can punish individuals who attempt suicide [40]. The law states: “Whoever attempts to commit suicide and does any act towards the commission of such offense shall be punished with simple imprisonment for a term which may extend to one year, or with fine, or with both” [44]. Public education about suicide, its risk factors, prevention, and available helplines remain insufficient [45]. There is also a persistent lack of specialized clinical services for suicide prevention. Existing efforts in this area are limited, fragmented, and poorly coordinated [46, 47]. Often these efforts are dispersed, underfunded, or entirely unfunded, severely limiting their ability to create meaningful societal impact [36, 47].

## 5 Key Imperatives for Suicide Prevention in Bangladesh

In line with the SDG, national‐level support for suicide prevention is essential. Efforts should be guided by recommendations and evidence‐based practices from international documents and relevant sources to develop effective national strategies. At the policy level, the development of a national suicide prevention strategy should be prioritized to demonstrate the government's commitment to addressing suicide, identify critical evidence‐based actions, clarify responsibilities, and outline how various stakeholders should collaborate [16]. A multisectoral approach should be adopted and tailored to the country's unique sociocultural context [16]. Although developing such a strategy is complex and time‐consuming, requiring a systematic approach and clear action plans, several immediate priorities drawn from international guidelines are crucial for creating an effective policy framework or implementing practical measures to reduce suicide rates [48]. This review also outlines these priorities and assesses Bangladesh's readiness to implement suicide prevention initiatives.

### 5.1 Quality Suicide Data

Due to the absence of a routine national suicide surveillance dataset, it is essential to establish such a national database to promote quality data [49]. The criminalization of suicide is an enabler to the underreporting and misclassification of suicides and contributes to lower WHO estimates [4]. Under the existing legal framework, the police and law enforcement agencies serve as the primary responders to suicide deaths, with each police station maintaining records of such incidents. A standardized data collection template, developed in consultation with experts and managed by trained personnel at each police station, could be a potential solution for capturing accurate suicide data. With the current data collection infrastructure in place, establishing a routine and integrated surveillance system would require only minor structural adjustments [32]. Overall, the system should link local police stations, district offices, and national headquarters, ensuring accessibility for the criminal justice system, health services, and other relevant stakeholders. Additionally, nationally representative surveys focusing on suicidal behavior are needed to provide a situational analysis of the risk and protective factors and to identify the vulnerable groups within their specific context [14, 16]. Currently, suicide‐related research in Bangladesh remains limited. No nationwide, longitudinal, or interventional studies have explored the biopsychosocial dimensions of suicide [4].

### 5.2 Responsible Media Reporting on Suicide

Responsible media coverage is vital for effective suicide prevention [18]. Instead of sensationalizing or glamorizing suicide cases, which can trigger imitation among at‐risk individuals, media professionals should highlight stories of resilience, recovery, and help‐seeking. The media has also the power to raise awareness about suicide and its prevention, foster mental health education, and support efforts to reduce societal stigma. Actively engaging media outlets in the development, dissemination, and training of responsible reporting practices is essential for improving the quality of suicide coverage and minimizing the risk of imitation [14, 16]. In this context, the Ministry of Information should issue clear directives requiring all media outlets to adhere to WHO guidelines for reporting suicide which has been rarely supported by media professionals [42]. Academic institutions that train journalists should integrate coursework on ethical and responsible suicide reporting into their curricula. At the same time, media organizations should conduct training and awareness programs for professionals at both national and local levels [42].

### 5.3 Means Restriction

Limiting access to common methods of suicide is an effective prevention strategy, as it reduces the risk of impulsive suicides, providing vulnerable individuals with more time to reconsider their actions [50]. Such strategies can be implemented nationally through laws and regulations and locally by making high‐risk environments safer [16]. As noted earlier, pesticide ingestion and hanging are the two most common methods of suicide and suicide attempts in Bangladesh [4, 40]. As an agrarian country, it is critical for Bangladesh to implement measures that restrict access to pesticides and other highly lethal means, especially in areas where vulnerable populations are most at risk [51]. Without strict monitoring of pesticide availability and distribution, suicide prevention efforts will be severely compromised [52]. Sri Lanka offers a successful example, achieving a 70% reduction in its overall suicide rate over two decades through means restriction, primarily via government regulations and successive bans on HHPs in agriculture [53, 54]. Bangladesh could benefit from such best practices by strengthening the regulation and management of pesticide use [13]. However, limited enforcement capacity and weak rural regulations may hinder progress. Implementing and testing safe pesticide storage practices in Bangladesh could be beneficial. Further research is needed to identify and develop culturally sensitive measures [55, 56].

### 5.4 Decriminalization of Suicidal Attempt

Although debates persist regarding the criminalization versus decriminalization of suicidal behavior, many researchers argue that decriminalization may reduce barriers to help‐seeking, lessen fears of legal repercussions, and encourage more accurate reporting of suicide attempts [36]. Criminalization can discourage individuals experiencing suicidal crises from disclosing their distress or accessing healthcare services, while also contributing to underreporting and incomplete surveillance data [57, 58]. From a public health perspective, decriminalization reframes the response to suicidal behavior from legal enforcement toward prevention, treatment, and support. However, decriminalization alone is unlikely to reduce suicide rates without concurrent investments in mental health services, crisis intervention, public awareness campaigns, and community‐based prevention programs. Consequently, decriminalization should be viewed as one component of a comprehensive suicide prevention strategy rather than a standalone solution.

Recently, Malaysia, a Muslim‐majority country, decriminalized suicide as part of a broader strategy to address suicidal behavior through healthcare and social support systems instead of criminal penalties. However, it is difficult to assess the immediate impact of this policy intervention, as the act only came into force in September 2025. In Bangladesh, attitudes toward decriminalization remain an important consideration. A study of Bangladeshi Imams (religious leaders) found that 50.6% believed that decriminalization would not help prevent suicide [59]. Although this finding highlights potential concerns among religious leaders, it reflects the views of a specific stakeholder group and cannot be generalized to the broader population. However, the decision to decriminalize suicide may not be straightforward for a Muslim‐majority country like Bangladesh. For example, although Pakistan, an Islamic republic, officially decriminalized suicide in December 2022, it recently reinstated criminal penalties for suicide attempts on religious grounds. Therefore, further research is needed to understand how decriminalization is perceived by healthcare professionals, policymakers, individuals with lived experience, and the general public. Such evidence would inform policy decisions and clarify how decriminalization could contribute to a broader, evidence‐based suicide prevention strategy in Bangladesh.

### 5.5 Community‐Based Suicide Prevention

Although the WHO emphasizes the central role of governments in suicide prevention, it also highlights the importance of local communities and other stakeholders in implementing effective actions [17]. Developing effective suicide prevention strategies at the rural or local level is crucial for reducing national suicide rates [60]. Local institutions play a vital role in implementing national suicide prevention plans and can also develop and tailor interventions for regions where national strategies are lacking [61].

Although no nationwide survey has been conducted, available evidence suggests that the southwestern region of Bangladesh, particularly the districts of Chuadanga, Jhenaidah, Kushtia, and Meherpur, has the highest prevalence of suicide [4]. For instance, official records show that between 2010 and 2018, Jhenaidah alone reported 3152 suicides and 22,675 suicide attempts [13]. The higher suicide rates in Jhenaidah include a complex interplay of financial difficulties, psychological and physical distress, relationships and family conflict, educational and social pressures, accessible to lethal means, and stigma surrounding mental illness [62]. Accurate determination of area‐wise concentrations could enable targeted community‐based interventions, which may be the most effective approach for suicide prevention. For example, Societies for Voluntary Activities (SOVA), a Bangladeshi local NGO, played a pivotal role in Jhenaidah by launching community interventions that greatly enhanced public understanding of suicide and self‐harm. However, the organization is currently nonfunctional due to funding constraints [36, 47]. Extensive research on suicide‐prone areas, such as Jhenaidah, is lacking. Consequently, there is a scarcity of measurable findings on recent trends in suicide rates and local risk factors. Strengthening the capacity of local NGOs could enable them to implement community‐level suicide prevention initiatives with greater effectiveness.

### 5.6 School‐Based Suicide Prevention Programs

Youth are at particular risk for suicide [1, 8]. Suicide was identified as the third leading cause of death among individuals aged 15–29 years and the fourth leading cause of death among 15–19‐year‐olds, for both sexes [1]. The risk factors are multifaceted and include substance use, childhood abuse, stigma against help‐seeking, barriers to accessing care, and access to means of suicide [63]. Early detection and customized interventions are crucial for reducing suicide risk and offering meaningful support to affected children, adolescents, and their families [64]. Schools serve as ideal environments for identifying and preventing suicide, as students spend a significant portion of their time in these settings [65]. The WHO's [18] *LIVE LIFE: An Implementation Guide for Suicide Prevention in Countries* emphasizes the importance of developing adolescents’ socio‐emotional life skills through school‐based interventions. Further recommendation includes the integration of student‐focused programs with staff training on identifying risk factors and warning signs, offering support to distressed youth, and referring them to appropriate services when necessary [66]. School‐based suicide prevention programs comprise several key components: gatekeeper training for staff, curriculum‐based education for students, and screening processes to detect at‐risk individuals [65]. However, these programs vary considerably in scope, content, cost, target audience, duration, and delivery methods [66]. Although the Bangladeshi *National Adolescent Health Strategy 2017–2023* was developed in response to the SDGs to promote mental health through interventions and screening [67], no concrete interventions have been implemented. Further research is needed to identify culture‐specific, school‐based preventive measures that align with global guidelines and evidence. Cost‐effectiveness and persistent stigma may pose significant challenge to implementing school‐based suicide prevention programs. Nevertheless, these approaches could be piloted as components of broader school‐based mental health services.

### 5.7 Resource Mobilization for Suicide Prevention

No intervention is possible without sufficient financial resources. The WHO's [18] *LIVE LIFE: An Implementation Guide for Suicide Prevention in Countries* highlights that suicide prevention is often undervalued as a public health priority, resulting in limited dedicated funding. Integrating suicide prevention efforts with other sectors can create opportunities for solution‐driven, cross‐sectoral funding. Funding for suicide prevention may be allocated at different levels: geographic (national or local), stakeholder (such as schools or the agricultural sector), and system‐wide (including research, technology, and capacity building). Bangladesh currently demonstrates a lack of clear commitment and action in this area. Funding for suicide prevention, research, and mental health services is extremely limited. For instance, the national budgetary allocation for mental health represents about only 0.50% of the total health budget [36, 41]. The *Bangladesh Mental Health Strategic Plan* (2022) has emphasized the importance of allocating sufficient finances for mental health, which could positively impact suicide prevention efforts [41]. Although underfunding poses a significant challenge, strong political commitment can mitigate this problem. The government needs to prioritize suicide prevention on its policy agenda and encourage national and international donors, corporate organizations, and individual benefactors to support suicide prevention initiatives. Above all, a dedicated government budget allocation for suicide prevention is the most critical requirement to ensure sustained and effective action. Transitioning suicide prevention from a predominantly mental health framework to a comprehensive public health model has been recommended. This shift could help secure dedicated funding, enhance multisectoral collaboration, improve surveillance and prevention systems, and fully integrate suicide prevention into national health and development priorities. This approach would frame suicide as a public health challenge at the population level, demanding coordinated action among healthcare, education, social welfare, law enforcement, and community sectors.

## 6 Conclusion

The SDGs serve as a vital framework for tracking global progress in mental health and suicide prevention. Although Bangladesh currently faces significant gaps in suicide prevention initiatives, there remains a significant opportunity for meaningful and timely action. By adopting international best practices and tailoring strategies to local needs and cultural contexts, the country can make measurable progress in reducing suicide and supporting vulnerable populations.

To achieve this, Bangladesh should formulate a comprehensive and collaborative national suicide prevention strategy, accompanied by targeted, evidence‐based interventions that address the specific needs of different communities and at‐risk groups. Strengthening collaboration among government agencies, healthcare providers, educational institutions, civil society organizations, and community leaders can foster a more integrated and effective response.

Despite the profound social, economic, and public health consequences, suicide prevention remains severely under‐prioritized in Bangladesh. Addressing this challenge demands sustained political commitment, adequate resource allocation, enhanced surveillance and data collection, expanded access to mental health services, and ongoing public awareness campaigns. With long‐term investment and collective action, Bangladesh can progress toward achieving SDG Target 3.4, lower suicide mortality rates, and create a safer, more supportive environment that fosters mental well‐being and resilience for all.

## Author Contributions

*Conceptualization, project administration, and supervision:* Anisur Rahman Khan. *Writing – original draft preparation and writing – review and editing:* all authors. All authors have read and approved the final version of the manuscript.

## Funding

The authors have nothing to report.

## Ethics Statement

All data were drawn from public sources. No personally identifiable information was disclosed. Ethical clearance was not required for public domain analysis.

## Conflicts of Interest

The authors declare no conflicts of interest.

## Data Availability

Data sharing is not applicable to this article as no datasets were generated or analyzed during the current study.
